# Dietary hot red pepper powder modulates laying performance, hematology profile, and hepatic antioxidant status in laying hens under tropical conditions

**DOI:** 10.1016/j.vas.2026.100891

**Published:** 2026-10-06

**Authors:** Muhammad Fathin Hanif, Ali Agus, Bambang Ariyadi, Muhlisin, Nanung Danar Dono, Heru Sasongko, Chusnul Hanim

**Affiliations:** a Department of Animal Production, Faculty of Animal Science, Universitas Gadjah Mada, Jalan Fauna No. 3, Yogyakarta, 55281, Indonesia; b Department of Animal Nutrition and Feed Science, Faculty of Animal Science, Universitas Gadjah Mada, Jalan Fauna No. 3, Yogyakarta, 55281, Indonesia

**Keywords:** Hot red pepper, Laying hens, Oxidative stress, Liver health, Laying performance

## Abstract

Hot red pepper (HRP) has emerged as a phytogenic feed additive with the potential to enhance laying performance in laying hens by improving gut health and mitigating oxidative stress. This study aimed to evaluate the effects of dietary inclusion of HRP powder on the productive performance, hematological and biochemical profiles, intestinal morphology, oxidative stress markers, and liver histopathology of laying hens reared under tropical conditions. A total of 100 Novogen laying hens were randomly assigned to four dietary treatments for eight weeks: a basal control diet and diets supplemented with 0.25%, 0.5%, or 0.75% HRP powder. Dietary supplementation with 0.25% and 0.5% HRP improved hen-day egg production during the first period (P < 0.05). HRP inclusion significantly reduced serum cholesterol levels (P < 0.05). Additionally, HRP supplementation (0.25–0.75%) increased lymphocyte counts (P < 0.001) while decreasing total leukocytes, heterophils, and the heterophil-to-lymphocyte (H/L) ratio (P < 0.001). Regarding gut environment, dietary inclusion of 0.5%-0.75% HRP decreased caecal pH, whereas 0.75% HRP reduced colon pH (P < 0.05). For antioxidant status, HRP at 0.25–0.5% enhanced hepatic superoxide dismutase activity (P < 0.05), while all inclusion levels (0.5–0.75%) lowered malondialdehyde concentrations (P < 0.05). Histopathological evaluation confirmed reduced liver inflammation and cellular degeneration. Overall, dietary HRP improves productive performance, physiological status, gut function, and liver health in laying hens under tropical conditions, with 0.5% providing the most consistent benefits, supporting its application as an effective phytogenic feed additive in poultry production.

## 1 Introduction

As global demand for poultry products continues to rise, modern poultry production systems increasingly require high growth performance and productivity, thereby driving the ongoing intensification of genetic selection and improvement programs. However, selection strategies primarily oriented toward enhancing production performance have inadvertently increased poultry susceptibility to environmental stressors, particularly under tropical conditions (Attia et al., 2024; Oke et al., 2024; Onagbesan et al., 2023). This trend is evidenced by the marked improvement in laying hen productivity, which has increased from approximately 150 eggs per hen per year in the early 1900s to around 300 eggs annually in modern commercial strains (American Egg Board, 2020). Broilers and laying hens developed through intensive genetic selection exhibit elevated metabolic rates and production efficiency (Tallentire et al., 2016), leading to increased endogenous heat production and greater susceptibility to physiological stress (Goo et al., 2019). Consequently, rapid metabolism rate may serve as a potential source of pro-oxidants and oxidative stress (Rafiei & Khajali, 2021).

Commercial broilers and laying hens reared in tropical climates often face challenges in reaching their full genetic potential due to suboptimal environmental conditions (Oke et al., 2024; Soliman & Safwat, 2020). Modern commercial laying hens, which have been intensively selected for high egg production, are particularly vulnerable to heat stress as a consequence of elevated metabolic heat generation (Narinç et al., 2016). This vulnerability is further aggravated by anatomical and physiological constraints, such as the absence of sweat glands and dense feather coverage that limit efficient heat dissipation (Hester, 2017; Hu et al., 2021; Lin et al., 2006). Moreover, comparative evaluations between indigenous and commercial chicken genotypes indicate that commercial strains exhibit lower expression of heat shock proteins (HSPs), which is associated with reduced heat tolerance and increased susceptibility to oxidative damage (Cedraz et al., 2017; Sadr et al., 2023).

Beyond thermal stress, chickens may also experience physiological stress arising from multiple external factors, including environmental fluctuations, toxin exposure, and pathogenic infections (Abbas et al., 2020; Emami et al., 2020; Gu et al., 2022b; Liu et al., 2021b). Among these stressors, heat stress constitutes a major constraint in poultry production, particularly in tropical regions where elevated ambient temperature and humidity markedly impair egg production and physiological stability (Jongbo, 2020; Tesakul et al., 2025; Varguez-Montero et al., 2012). Prolonged exposure to extreme thermal conditions disrupts normal physiological functions, resulting in decreased laying performance, compromised health status, and increased mortality. One of the principal biological consequences of heat stress is the induction of oxidative stress, which critically affects reproductive performance, immunity, and overall productivity in laying hens. Oxidative stress (OS) develops when the generation of reactive oxygen species exceeds the capacity of endogenous antioxidant defense systems, thereby causing cellular redox imbalance (Chen et al., 2024; Lin et al., 2025; Oke et al., 2024). This imbalance can subsequently damage essential biomolecules—including lipids, deoxyribonucleic acid (DNA), and proteins—leading to impaired cellular function and contributing to the development of pathological conditions (Ďuračková, 2010; Mesa-Garcia et al., 2018).

OS adversely affects laying hens by impairing laying performance, egg quality, and immune function (Kim & Lee, 2023). At the reproductive level, OS accelerates ovarian aging, induces follicular atresia, and promotes granulosa cell apoptosis, thereby directly reducing egg production and quality (Yang et al., 2024). Metabolically, OS can decrease feed intake and disrupt energy metabolism, further compromising overall productivity (Zhou et al., 2020). In addition, OS exerts detrimental effects on vital organs, particularly the liver, where it contributes to hepatic damage, lipid accumulation, and inflammatory responses (Miao et al., 2023; Wang et al., 2021). From an immunophysiological perspective, OS alters leukocyte dynamics by increasing heterophil counts—key components of the innate immune response—while reducing lymphocyte levels. This imbalance results in an elevated heterophil-to-lymphocyte (H:L) ratio, which is widely recognized as an indicator of stress-induced immune modulation (Keten & Matur, 2022; Kim et al., 2021; Matur et al., 2015).

HRP, which originated from tropical regions and requires high temperatures for vegetative growth and fruit development, produces fruits of *Capsicum annuum*, commonly known as chili, that have long been used as vegetables, flavor enhancers, natural colorants, and medicinal materials (Hernández‐Pérez et al., 2020; Oh & Koh, 2019; Saleh et al., 2021). HRP contains various bioactive compounds, including capsaicinoids, phenolic compounds, flavonoids, alkaloids, carotenoids, vitamin C, and vitamin E (Bae et al., 2012; Hernández‐Pérez et al., 2020; Montoya-Ballesteros et al., 2014). These constituents exhibit important biological activities, particularly antioxidant, anti-inflammatory, and antimicrobial properties (Bae et al., 2012; Hernández‐Pérez et al., 2020; Montoya-Ballesteros et al., 2014; Sanatombi, 2023). Consequently, HRP has attracted increasing attention as a potential natural feed additive in poultry production, with several studies reporting its beneficial effects on poultry performance, health status, and physiological responses (Abd El-Hack et al., 2022; Hanif et al., 2024).

Supplementation of HRP at 0.8% in the diet of laying hens for 2 weeks increased egg weight but did not affect laying performance, feed intake, or FCR (Li et al., 2012). Moreover, dietary HRP at 0.5% for 3 weeks did not affect productive performance or villus height and area, but increased cell area and mitotic activity in the jejunum of laying hens (Lokaewmanee et al., 2013). In contrast, HRP supplementation at 0.5% for 8 weeks improved laying performance and reduced cholesterol and MDA levels in both serum and yolk (Abou-Elkhair et al., 2018). In addition, supplementation of HRP at 0.5% for 10 weeks reduced feed intake but increased hen-day egg production and feed efficiency, as well as elevated serum IgG levels (Sözcü, 2019). Red pepper supplementation at 1% for 12 weeks improved hen-day egg production and egg mass, while reducing feed intake and FCR (Aikpitanyi & Imasuen, 2023). In addition, supplementation of RP at 1% for 12 weeks decreased white blood cell and heterophil counts, and increased lymphocyte levels of laying hens (Aikpitanyi & Imasuen, 2024). Moreover, supplementation of HRP at 1% improved ovarian weight of hens (Özer et al., 2005). However, a meta-analysis by Hanif et al. (2024) indicated that dietary inclusion of red pepper exceeding 0.9% leads to a reduction in feed intake.

In addition, capsaicin supplementation has been shown to enhance egg production rates (Zhang et al., 2025a, 2025b). Dietary capsaicin has also been reported to increase antioxidant enzyme activities, improve intestinal health, and suppress pro-inflammatory cytokines in laying hens (Fan et al., 2023; Zhang et al., 2025b). Capsaicin supplementation significantly enhances ovarian development in laying ducks by increasing the relative weight of large and small yellow follicles as well as the number of dominant follicles. At the molecular level, capsaicin upregulates mRNA expression related to calcium signaling and cell cycle regulation. Furthermore, capsaicin strengthens the antioxidant system by increasing plasma glutathione peroxidase activity and reducing MDA levels in ovarian tissue (Liu et al., 2021a).

Although bioactive compounds in HRP possess antioxidant and anti-inflammatory properties, their dose-dependent effects on laying hens under tropical conditions remain insufficiently understood. Importantly, studies simultaneously evaluating laying performance, gut health, and liver histology under tropical conditions remain limited. Therefore, this study aimed to evaluate the effect of HRP powder on productive performance, hematological and biochemical profiles, liver antioxidant activity, and liver histopathology in tropical-reared laying hens. It was hypothesized that dietary HRP powder would dose-dependently enhance productive performance, antioxidant capacity, and physiological profiles, thereby alleviating oxidative stress while promoting gut health and liver histology.

## 2 Materials and methods

### 2.1 Animal ethic

The study protocol received ethical clearance from the Animal Research Ethics Committee of the Faculty of Veterinary Medicine, Universitas Gadjah Mada, Yogyakarta, Indonesia (Ref. No. 98/EC-FKH/Eks/2023).

### 2.2 Hot red pepper (HRP) powder sample

HRP samples were processed into powder through several steps. The peppers were first sorted to select good-quality fruits with a maturity level above 60%, healthy condition, and smooth physical appearance without defects. The stems and damaged parts were removed, and the selected peppers were washed to eliminate adhering dirt and then drained. After blanching, drying was carried out using an oven dryer at 60 °C until the moisture content reached below 9% (approximately 7–8%), with a drying duration of 10 h. The dried peppers were subsequently ground using a hammer mill equipped with a 60-mesh sieve to obtain a fine and homogeneous HRP powder for further analysis. The bioactive compounds of the HRP powder are presented in Table 2.

### 2.3 Hen, experimental diet, and housing

A total of 100 Novogen strain laying hens aged 56 weeks (body weight: 1.83 ± 0.054 kg; daily egg production: 83.24% ± 2.80) were randomly allocated to four dietary treatments in a completely randomized design, with five replicates per treatment and five hens per replicate. The feeding trial lasted for eight weeks, from 56 to 64 weeks of age. The basal diet consisted of corn, rice bran, soybean meal, crude palm oil, DL-methionine, L-lysine, limestone, dicalcium phosphate, mineral premix, vitamin premix, and choline chloride (Table 1). The dietary treatments included C0 (control, basal diet), C1 (basal diet + 0.25% HRP powder), C2 (basal diet + 0.5% HRP powder), and C3 (basal diet + 0.75% HRP powder) were selected based on the dosage range mapping reported in the meta-analysis by Hanif et al. (2024). The antioxidant activity of the diets increased with the addition of HRP powder, as shown in Fig. 1**.** Hens were provided feed in coarse mash form at 120 g/hen/day, while drinking water was supplied ad libitum.

**Table 1:** Feed ingredients and nutrient composition of the experimental diet.

**Ingredient (%)**	**C0 (Control)**	**C1 (HRP 0.25%)**	**C2 (HRP 0.50%)**	C3 (HRP 0.75%)
Yellow corn	56.65	56.65	56.65	56.65
Rice bran	3.60	3.60	3.60	3.60
Soybean meal	24.80	24.80	24.80	24.80
Limestone	10.00	10.00	10.00	10.00
Crude palm oil	1.50	1.50	1.50	1.50
Dicalcium phosphate	1.75	1.75	1.75	1.75
DL-methionine	0.25	0.25	0.25	0.25
L-lysine	0.10	0.10	0.10	0.10
Common salt	0.25	0.25	0.25	0.25
Mineral mix^a^	0.20	0.20	0.20	0.20
Vitamin mix^b^	0.05	0.05	0.05	0.05
Choline chloride	0.10	0.10	0.10	0.10
Hot red pepper powder	0.00	0.25	0.50	0.75
Filler	0.75	0.50	0.25	0.00
Total	100.00	100.00	100.00	100.00
Calculated nutrient				
Metabolizable energy (kcal/kg)	2816	2816	2815	2815
Available phosphor (%)	0.42	0.42	0.42	0.42
Methionine (%)	0.52	0.52	0.52	0.52
Lysine (%)	1.07	1.07	1.07	1.07
Methionin + Cysteine (%)	0.82	0.82	0.82	0.82
Threonine (%)	0.68	0.68	0.68	0.68
Analyzed nutrient				
Gross energy (kcal/kg)	3488	3472	3488	3519
Dry matter^c^ (%)	90.32	90.27	89.51	90.22
Crude protein^c^ (%)	18.10	18.08	18.11	18.15
Ether extract^c^ (%)	4.77	4.22	4.20	4.23
Crude fiber^c^ (%)	4.53	4.18	4.72	4.82
Calcium^c^ (%)	5.28	5.61	5.12	5.31
Total phophor^c^ (%)	0.65	0.62	0.60	0.61

Note:.

a Content per kg: sodium 29.3 g; calcium 261.5 g; iron 17.7 g; potassium 2.4 g; magnesium 2.5 g; phosphorus 6.9 g; sulfur 783.6 μg; manganese 900 μg; copper 186.6 μg; zinc 361.6 μg; selenium 0.61 μg; and cobalt 5.3 μg.

b Content per kg: vitamin D₃ 4000,000 IU; vitamin A 20,000,000 IU; carnitine 20,000 mg; vitamin K₃ 7000 mg; vitamin B₂ 12,000 mg; vitamin B₁₂ 60 mg; vitamin E 15,000 mg; vitamin B₆ 8000 mg; Ca-D-pantothenate 20,000 mg; niacin 40,000 mg; and folic acid 500 mg.

c Proximate composition was determined following AOAC (AOAC, 2005) procedures.

**Table 2:** Bioactive compounds of HRP (Capsicum annuum L.) powder.

Bioactive compound	Content
Capsaicin (%)	0.16 ± 0
Total phenolic compound (GAE, %)	5.7 ± 0.17
Total flavonoid (QE^1^, %)	1.0 ± 0.11
Alkaloid (QE^2^, %)	0.31 ± 0.01
Saponin (QS, %)	3.1 ± 0.20
Carotene (mg/100 g)	79.0 ± 1.7
Xanthophyll (mg/100 g)	99.8 ± 2.3
Vitamin C (mg/100 g)	327 ± 2.2
Vitamin E (mg/100 g)	39.5 ± 1.3

Note: GAE: gallic acid equivalent, QE^1^: quercetin equivalent, QE^2^: quinine equivalent, QS: quillaja saponin, TAE: tannic acid equivalent.

**Fig. 1:**
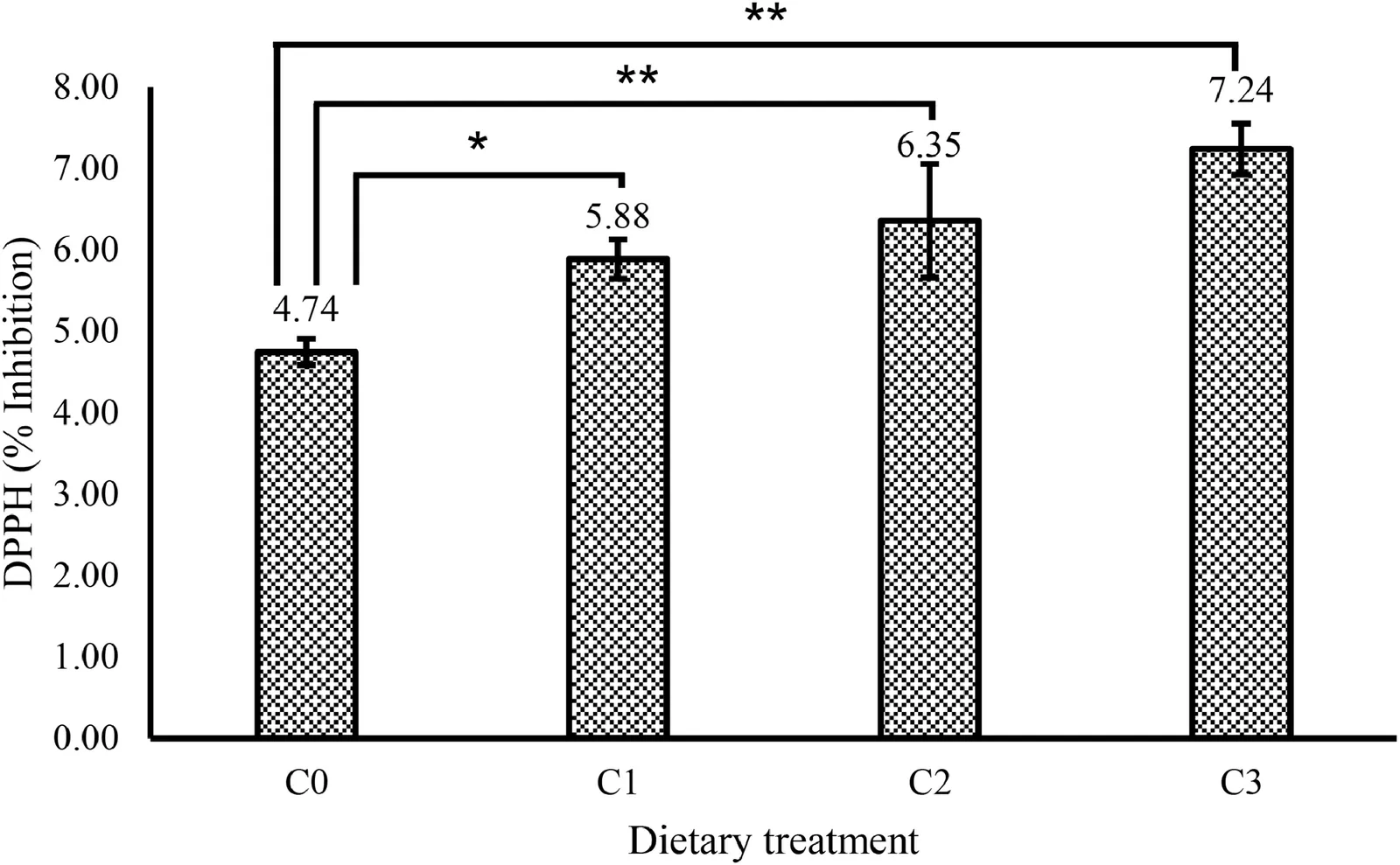
Antioxidant activity (DPPH) of diets supplemented with HRP powder.

**Note:** C0, basal diet; C1, basal diet + 0.25% HRP powder; C2, basal diet + 0.5% HRP powder; C3, basal diet + 0.75% HRP powder; *, significant at P < 0.05; **, significant at P < 0.01.

The laying hens were housed individually in galvanized battery cages arranged in a three-tier system. Each cage measured 22 cm in length, 35 cm in width, and 30 cm in height, providing a stocking density of 0.077 m² per hen (Fig. 2). Each cage was equipped with a feeder and a nipple drinker. To ensure continuous access to clean drinking water, one nipple drinker was installed in each cage. Lighting inside the housing facility was managed to support the hens’ physiological rhythm using warm-white LED lamps operated for 16 h daily, from 06:00 to 22:00. Laying hens were housed in an open-sided barn owned by PT Agro Mix Lestari, located in Banaran Kidul Village, Sentolo District, Kulon Progo Regency, Special Region of Yogyakarta, Indonesia. The study site (7°49′03.82″ S; 110°12′32.34″ E; 79.01 m above sea level) is characterized by an annual rainfall of 1000–1500 mm (BMKG, 2024) and a mean daily temperature of 27.7 ± 0.9 °C. (CEIC, 2024). The barn was managed as a naturally ventilated open-house system without mechanical cooling devices such as fans or evaporative cooling, thereby reflecting typical tropical production conditions. Barn temperature and relative humidity were recorded hourly using an Elitech GSP-6 temperature–humidity data logger (Elitech, USA) throughout the rearing period (Fig. 3). The temperature–humidity index (THI) was calculated following Halder et al. (2023): THI = 0.8 × TA + RH × (TA − 14.4) + 46.4, where TA is the ambient temperature (°C) and RH is the relative humidity (%).

**Fig. 2:**
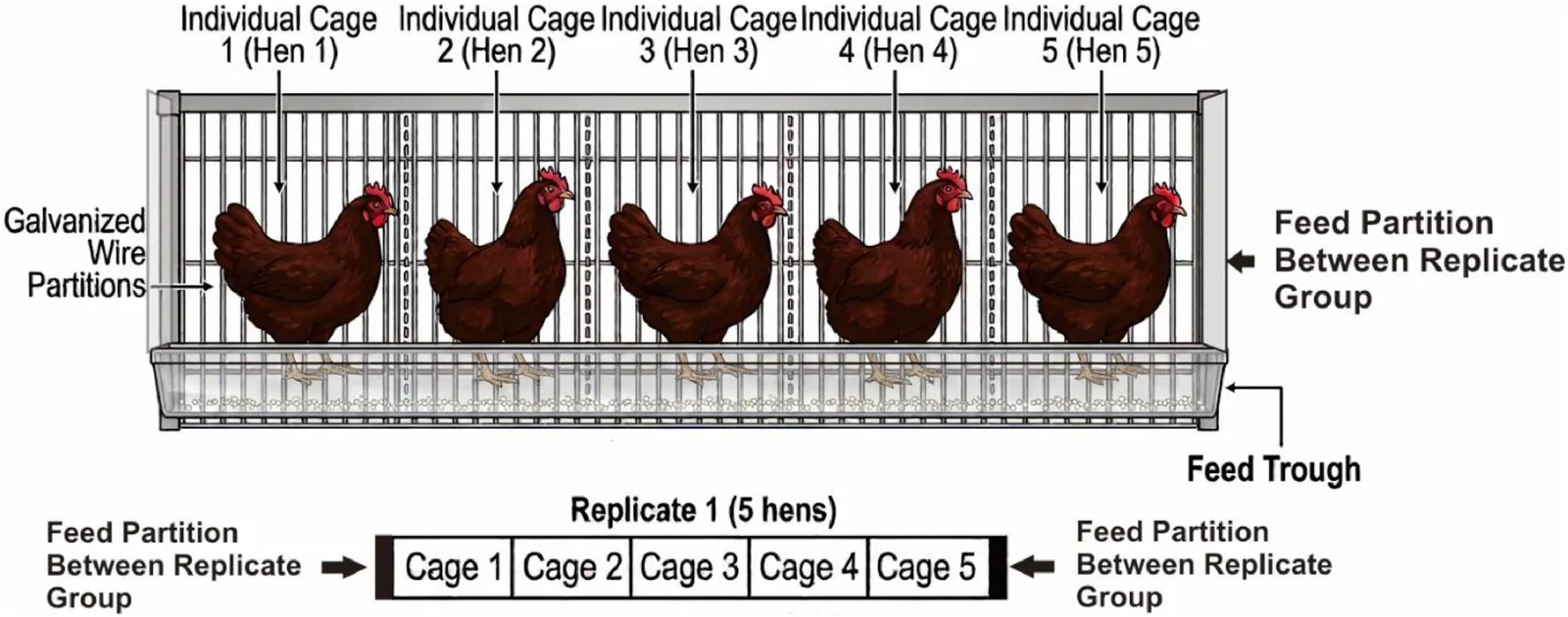
Experimental design and arrangement of individually housed laying hens within each replicate.

**Fig. 3:**
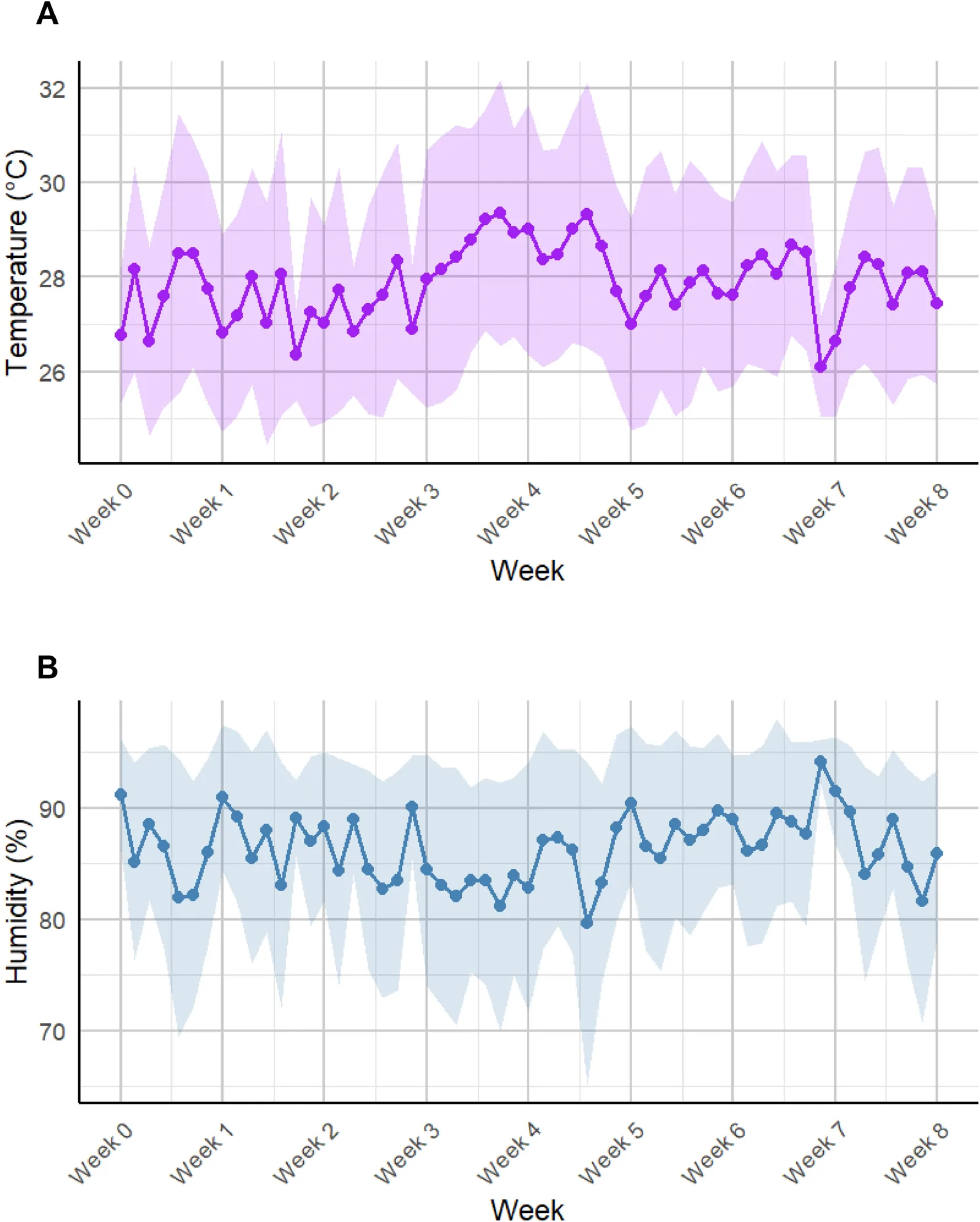

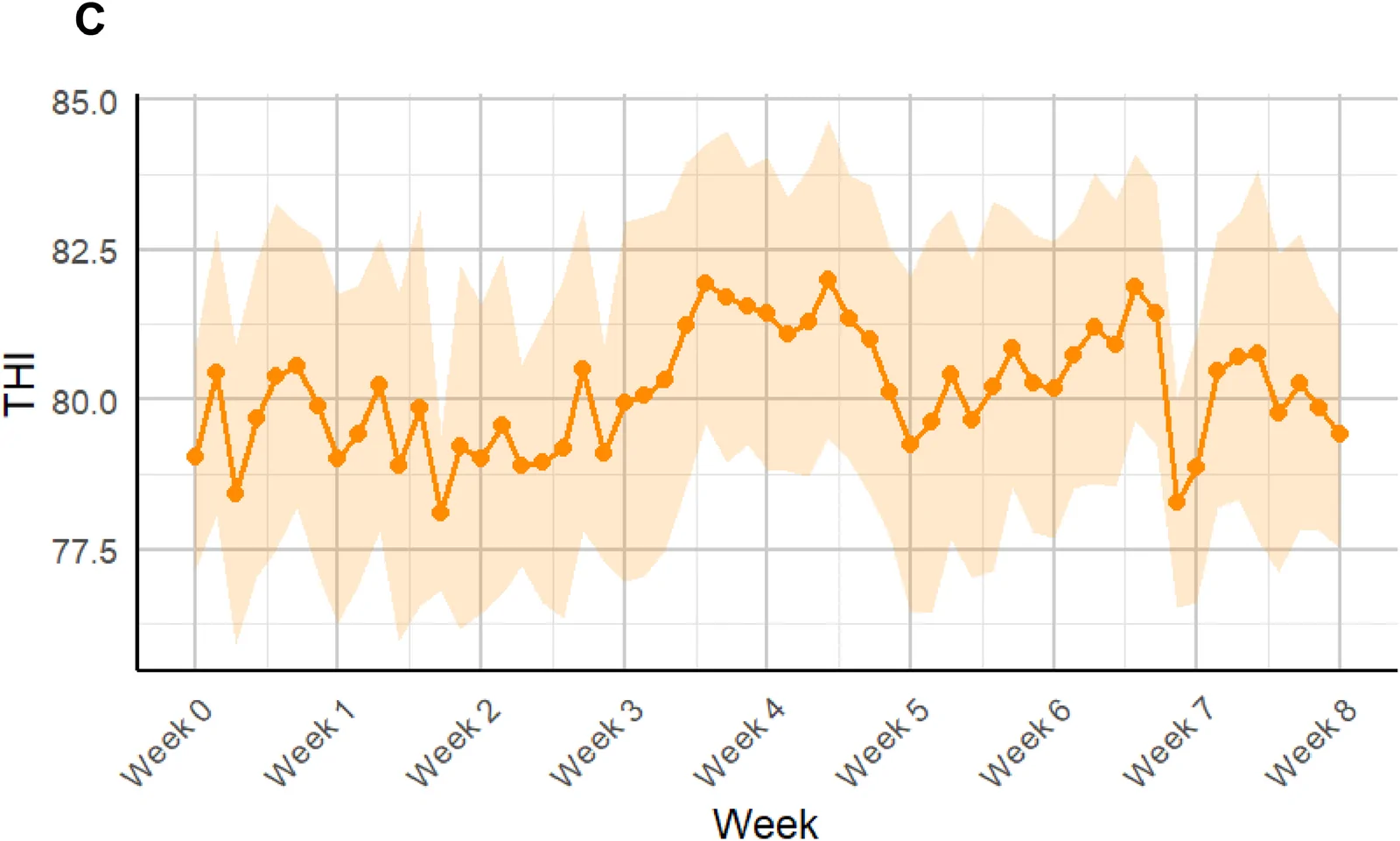
Environmental conditions in the laying hen house during the treatment period: (A) average temperature, (B) average relative humidity, and (C) average daily temperature–humidity index (THI).

Note: the average temperature and relative humidity were 27.9°C and 86.4%, respectively, with an average THI of 80.15, indicating a condition of moderate thermal discomfort in poultry (Moraes et al., 2008).

### 2.4 Laying performance

Laying performance was evaluated weekly at the replicate level (Fig. 2). Each experimental replicate consisted of five individually housed laying hens assigned to the same treatment, resulting in five replicates per treatment. The evaluated variables included hen-day egg production (HDA), egg weight (EW), egg mass (EM), feed intake (FI), and feed conversion ratio (FCR), following the procedure described by Anas et al. (2024). HDA was calculated as the total number of eggs produced within each replicate divided by the number of hens present, expressed as a percentage. EW was calculated by dividing total egg weight by the total number of eggs and expressed as grams per egg. EM was calculated as the total egg mass produced within each replicate, expressed on a per-hen basis, and standardized to 1000 hens per day. FI was determined as the difference between feed offered and feed refusals (orts), expressed as grams per hen. FCR was calculated as total feed intake divided by total egg mass produced within each replicate. The resulting weekly values from the five replicates per treatment served as the experimental units for statistical analysis. Income over feed cost (IOFC) was calculated in Indonesian Rupiah (IDR) as the difference between the average egg sales value and total feed cost during the entire feeding period, following Juan et al. (2024). Egg sales value was calculated by multiplying the mean number of eggs produced by the farm-gate price corresponding to each egg size category, whereas feed cost was determined by multiplying the average total feed intake by the cost per kilogram of diet (IDR/kg).

### 2.5 Blood hematological measurements

At the end of week 8, one hen with a body weight close to the median of each replicate was selected for blood sampling. Blood was collected from the wing vein (vena ulnaris superficialis) by a veterinarian following Shabbir et al. (2013) and transferred into EDTA-containing tubes (OneMed, Indonesia). Samples were stored at 1–4 °C for no longer than one week before hematological analysis. Hematological parameters were determined following Weiss and Wardrop (2010) and Adebisi (2017), with minor modifications, using a VETSCAN HM5 hematology analyzer (Abaxis, USA). The parameters included packed cell volume (PCV), total plasma protein (TPP), fibrinogen (FIB), hemoglobin (Hb), red blood cell (RBC) and white blood cell (WBC) counts, erythrocyte indices [mean corpuscular volume (MCV), mean corpuscular hemoglobin (MCH), and mean corpuscular hemoglobin concentration (MCHC)], and differential leukocyte percentages [monocytes (MON), lymphocytes (LYM), eosinophils (EOS), and heterophils (HET)].

### 2.6 Serum biochemical analysis

At the end of week 8 of treatment, one hen with body weight close to the median from each replicate group was selected for blood sampling. Blood was collected via the wing vein (vena ulnaris superficialis) by a veterinarian following Shabbir et al. (2013) protocols. Blood samples were placed in plain tubes (OneMed, Indonesia), centrifuged at 3000 rpm with refrigrated centrifuge (Eppendorf, Germany) for 15 min to obtain serum, and stored at −40 °C for up to one month before analysis. Serum biochemical parameters including albumin, total protein, urea, blood urea nitrogen (BUN), cholesterol, triglyceride, uric acid (UA), aspartate aminotransferase (AST/SGOT), alanine aminotransferase (ALT/SGPT), and alkaline phosphatase (ALP) were measured using validated photometric and enzymatic colorimetric assays with a Microlab 300 analyzer (Merck Vital Scientific, Darmslandt, Netherlands) suitable with the commercial kits (DiaSys Diagnostic System GmbH, Holzheim, Germany).

### 2.7 Slaughter procedure and visceral organ collection

At the end of week 8, one hen with body weight closest to the replicate group median was selected from each replicate group for slaughter. Birds were humanely euthanized by severing the trachea, esophagus, and major blood vessels, and death was confirmed before tissue collection. Reproductive organs were subsequently collected without defeathering to allow determination of relative reproductive organ weight. This procedure ensured accurate sampling of digestive and reproductive organs for further analysis. Visceral organs were carefully separated and sectioned according to anatomical regions, including the crop, proventriculus, gizzard, liver, kidneys, pancreas, ceca, duodenum, jejunum, ileum, and large intestine, as well as reproductive organs. After removal, digesta pH was first measured using a calibrated digital pH meter (Shenzhen Yago Technology Co., Ltd., China) by making a small incision in the gastrointestinal tract and inserting the electrode directly into the luminal contents. Subsequently, the organs were emptied of contents, cleaned, and weighed. Relative organ weight was calculated as empty organ weight divided by live body weight and expressed as a percentage.

Reproductive organs were carefully separated and measured according to their anatomical components. The observed reproductive structures included the ovary—comprising the largest (first) follicle and the total number of follicles—as well as the oviduct, which was divided into the infundibulum, magnum, isthmus, uterus, and vagina. After separation, the organs were cleaned of residual contents to prevent contamination and subsequently weighed. Relative organ weight was calculated by dividing the cleaned organ weight by live body weight and expressing the result as a percentage.

### 2.8 Liver histopathology

One liver tissue sample was collected from each slaughtered hen (1 hen per replicate group, n = 5 per treatment). Liver tissue samples were fixed in 10% buffered formalin (100 mL of 40% formaldehyde, 4 g of monobasic sodium phosphate, 6.5 g of dibasic sodium phosphate, and 900 mL of distilled water), with tissue thickness adjusted to ensure adequate fixative penetration. Following fixation, tissues were dehydrated through a graded alcohol series, cleared using xylol, and infiltrated with molten paraffin before being embedded into paraffin blocks and allowed to solidify. Paraffin blocks were sectioned using a microtome (Leica Microsystems Ltd., Wetzlar, Germany) at a thickness of 2–5 µm to prepare four tissue sections per sampled hen. The sections were floated on a warm water bath, mounted onto glass slides, incubated to promote tissue adhesion, deparaffinized in xylol, rehydrated through descending alcohol concentrations to water, and stained with hematoxylin and eosin (H&E). Subsequently, slides were dehydrated through graded alcohol, cleared in xylol, and permanently mounted with a suitable medium and coverslip.

Histopathological alterations in liver tissues were evaluated via descriptive qualitative examination following Hidayaturrohman et al. (2026). For each prepared slide, 5 to 10 non-overlapping microscopic fields per section were examined under a light microscope (Yazumi, China) at 10 × and 40 × magnifications, with photomicrographs captured using an Optilab camera and software (OptiLab, Indonesia). To ensure objective evaluation, the microscopic assessment was conducted by a pathologist who was blinded to the dietary treatment identities. Descriptive findings were documented based on the presence, structural extent, and patterns of hepatocyte cellular integrity, sinusoidal congestion, and inflammatory cell infiltration across all examined fields.

### 2.9 Jejunal histomorphology

One jejunum tissue sample was collected from each slaughtered hen (1 hen per replicate). A segment of jejunal tissue (2 cm) was fixed in formalin and processed using the same procedures as liver samples, then sectioned with a microtome (Leica Microsystems Ltd., Wetzlar, Germany). Jejunal morphology was examined at 40 × magnification using a light microscope (Yazumi, China) and documented with an Optilab digital camera (Optilab Indonesia). Morphometric analysis was performed with Image Raster Software version 4.0.5 (PT. Miconos Transdata Nusantara, Indonesia) to determine villus height (VH), crypt depth (CD), and the villus height-to-crypt depth ratio (VH/CD) following Ariyadi et al. (2019) procedures. Five villi from each bird were measured, and the average values were used for statistical analysis. After measurement, villus surface area (VSA) was calculated following El Sabry and Abd El-Ghany (2021).

### 2.10 Liver antioxidant activity

Liver samples were collected and immediately stored in vacuum-sealed plastic in a freezer at −40 °C and not kept for longer than one month prior to analysis. Superoxide dismutase (SOD) activity and malondialdehyde (MDA) concentration were determined using commercial assay kits (CheKine™ Micro Superoxide Dismutase Activity Assay Kit, KTB1030; and CheKine™ Micro Lipid Peroxidation (MDA) Assay Kit, KTB1050-EN; Abbkine, China) according to the manufacturer’s instructions.

### 2.11 Statistical analysis

All experimental data were statistically analyzed using IBM SPSS Statistics version 26.0 (IBM Corp., USA). The data were subjected to one-way analysis of variance (ANOVA), followed by Duncan’s multiple range test to identify significant differences among the four treatments. In addition, orthogonal polynomial contrast analysis was performed to determine the linear and quadratic trends in response to increasing treatment levels. Differences were considered statistically significant at P < 0.05.

## 3 Results

### 3.1 Laying performance

The effects of dietary HRP powder inclusion on laying performance are presented in Table 3. Supplementation with 0.25% and 0.5% HRP powder increased HDA during period 1 (P < 0.05). Furthermore, HDA increased linearly during period 2 (Linear, P = 0.02) and across the overall period (Linear, P = 0.03). In addition, HRP supplementation linearly increased EM during period 2 (Linear, P = 0.04), and the overall period (Linear, P = 0.04). In addition, dietary inclusion of HRP powder linearly decreased FCR during period 2 (Linear, P = 0.04), and the overall period (Linear, P = 0.04). However, supplementation with HRP powder had no significant effect on FI, EW, or IOFC throughout the experimental periods (P > 0.05).

**Table 3:** Effect of HRP powder supplementation on the laying performance of laying hens aged 56–64 weeks (n = 5).

Variable	Group treatment				SEM	*p*-value (combined)	Contrasts	
	C0 (Control)	C1 (0.25%)	C2 (0.50%)	C3 (0.75%)			Linear	Quadratic
**56–60 weeks**								
FI (g)	111.6	112.4	112.2	111.8	0.19	0.48	0.78	0.15
HDA (%)	80.3^b^	82.2^a^	82.6^a^	81.3^ab^	0.34	<0.05	0.22	0.01
EW (g)	59.3	58.8	60.3	60.2	0.36	0.44	0.23	0.81
EM (g)	47.8	48.2	49.5	49.6	0.38	0.20	0.11	0.25
FCR	2.3	2.3	2.3	2.2	0.02	0.23	0.10	0.46
IOFC (Rp/bird)	221	209	214	187	8.8	0.16	0.08	0.25
**61–64 weeks**								
FI	111.4	111.6	111.2	110.9	0.15	0.43	0.17	0.49
HDA (%)	79.6	79.6	80.7	82.8	0.54	0.10	0.02	0.31
EW (g)	59.5	60.1	60.9	60.4	0.40	0.50	0.26	0.40
EM (g)	47.4	47.9	49.2	50.0	0.54	0.23	0.04	0.94
FCR	2.4	2.3	2.3	2.2	0.03	0.20	0.03	0.89
IOFC (Rp/bird)	212	201	206	200	11.7	0.99	0.79	0.94
**56–64 weeks**								
FI (g)	111.5	112.0	111.7	111.4	0.11	0.26	0.49	0.09
HDA (%)	79.9	80.9	81.7	82.0	0.35	0.15	0.03	0.65
EW (g)	59.3	59.5	60.6	60.3	0.35	0.52	0.22	0.72
EM (g)	47.4	48.1	49.5	49.5	0.41	0.19	0.04	0.63
FCR	2.3	2.3	2.3	2.3	0.02	0.17	0.04	0.75
IOFC (Rp/bird)	217	207	214	185	9.4	0.66	0.33	0.63

Note: HDA, hen-day egg average production; FI, feed intake; EW, egg weight; EM, egg mass; FCR, feed conversion ratio. ^a,b^ Different superscripts within the same row indicate a significant difference (P < 0.05). C0, basal diet; C1, basal diet + 0.25% hot red pepper powder; C2, basal diet + 0.50% hot red pepper powder; C3, basal diet + 0.75% hot red pepper powder. SEM, standard error of the mean.

### 3.2 Blood biochemical profile

The effects of HRP powder supplementation on the serum biochemical variables of laying hens were presented in Table 4. Supplementation with 0.25%, 0.5%, and 0.75% HRP powder did not affect total protein, albumin, glucose, triglyceride, urea, blood urea nitrogen, uric acid, and alkaline phosphatase (P > 0.05). However, dietary inclusion of HRP powder at 0.25%, 0.5%, and 0.75% reduced serum cholesterol concentrations (Combined, P = 0.04; Linear, P = 0.01; Quadratic, P = 0.09). In addition, a quadratic effect was observed for serum SGOT activity (Quadratic, P = 0.03).

**Table 4:** Effects of dietary hot red pepper powder supplementation on the serum biochemical parameters of laying hens (n = 5).

Variable	Group treatment				SEM	*p*-value (combined)	Contrasts	
	C0 (Control)	C1 (0.25%)	C2 (0.50%)	C3 (0.75%)			Linear	Quadratic
Total protein (g/dl)	4.8	5.0	5.1	4.9	0.12	0.86	0.77	0.42
Albumin (g/dl)	2.6	2.6	2.6	2.6	0.07	0.99	0.86	0.94
Glucose (mg/dl)	218	218	219	218	1.5	0.99	0.99	0.86
Cholesterol (mg/dl)	136**^a^**	114**^b^**	107**^b^**	110**^b^**	0.42	0.04	0.01	0.09
Triglyceride (mg/dl)	1011	752	862	788	51.8	0.32	0.24	0.38
Urea (mg/dl)	3.0	3.0	3.0	3.1	0.07	0.95	0.83	0.63
BUN (mg/dl)	1.5	1.6	1.5	1.5	0.07	0.91	0.82	1.0
Uric acid (mg/dl)	6.5	3.9	5.6	5.2	0.44	0.24	0.61	0.22
SGOT (U/I)	216	174	164	189	7.9	0.08	0.16	0.03
SGPT (U/I)	12.4	6.7	7.9	7.4	0.85	0.06	0.06	0.10
ALP (U/I)	630	500	463	434	67.3	0.77	0.34	0.72

Note: SGOT, serum glutamic oxaloacetic transaminase; SGPT, serum glutamic pyruvic transaminase; ALP, alkaline phosphatase. a–c Different superscripts within the same row indicate significant differences (P < 0.05). C0, basal diet; C1, basal diet + 0.25% hot red pepper powder; C2, basal diet + 0.50% hot red pepper powder; C3, basal diet + 0.75% hot red pepper powder. SEM, standard error of the mean.

### 3.3 Blood hematology

Dietary HRP supplementation did not significantly affect RBC, HB, erythrocyte characteristics (PCV, MCV, MCH, MCHC), TPP, or FIB (P > 0.05; Table 5). Dietary inclusion of HRP powder at 0.25%, 0.5%, and 0.75% reduced LEU counts (Combined, P < 0.001; Linear, P < 0.001; Quadratic, P < 0.001) and HET counts (Combined, P < 0.001; Linear, P < 0.001; Quadratic, P < 0.001). Furthermore, HRP supplementation at the same levels increased LYM counts (Combined, P < 0.001; Linear, P = 0.12; Quadratic, P < 0.001). Consequently, hens receiving 0.25%, 0.5%, and 0.75% HRP powder exhibited a lower H/L compared with the control group (Combined, P < 0.001; Linear, P < 0.001; Quadratic, P < 0.001).

**Table 5:** Effects of dietary HRP powder supplementation on the hematological parameters of laying hens (n = 5).

Variable	Group Treatment				SEM	*p*-value (combined)	Contrasts	
	C0 (Control)	C1 (0.25%)	C2 (0.50%)	C3 (0.75%)			Linear	Quadratic
RBC (10^6^/µl)	2.6	2.5	2.6	2.6	0.02	0.11	0.40	0.39
HB (g/dl)	11.4	11.6	10.4	10.9	0.33	0.65	0.40	0.89
PCV (%)	25.6	26.0	25.4	26.8	0.53	0.83	0.56	0.67
MCV (fL)	97.0	99.8	97.0	102.6	1.8	0.69	0.42	0.72
MCH (pg)	43.0	50.3	39.8	42.7	3.1	0.71	0.70	0.74
MCHC (pg)	44.7	48.7	41.0	41.0	3.0	0.80	0.52	0.75
TPP (g/dL)	6.7	6.8	6.9	7.8	0.37	0.75	0.36	0.60
FIB (g/dL)	0.34	0.22	0.16	0.36	0.53	0.51	1.0	0.16
LEU (10^3^/µl)	27.9^a^	21.7^d^	25.4^b^	23.7^c^	0.55	<0.001	<0.001	<0.001
HET (%)	41.6^a^	25.2^d^	29.0^c^	34.0^b^	1.5	<0.001	<0.001	<0.001
EOS (%)	1.0^c^	2.2^b^	2.8^a^	1.0^c^	0.19	<0.001	<0.001	<0.001
LYM (%)	53.6^c^	68.4^a^	64.0^b^	61.2^b^	1.3	<0.001	<0.001	<0.001
MON (%)	3.8	4.2	4.2	3.8	0.19	0.81	1.0	0.34
H/L	0.78^a^	0.37^d^	0.45^c^	0.56^b^	0.04	<0.001	<0.001	<0.001

Note: RBC, red blood cells; Hb, hemoglobin; PCV, packed cell volume (hematocrit); TPP, total plasma protein; MCHC, mean corpuscular hemoglobin concentration; FIB, fibrinogen; LEU, leucocytes; MON, monocytes; LIM, lymphocytes; EOS, eosinophils; MCV, mean corpuscular volume; MCH, mean corpuscular hemoglobin; H/L, heterophil-to-lymphocyte ratio. ^a–c^ Different superscripts within the same row indicate significant differences (P < 0.05). C0, basal diet; C1, basal diet + 0.25% hot red pepper powder; C2, basal diet + 0.50% hot red pepper powder; C3, basal diet + 0.75% hot red pepper powder. SEM, standard error of the mean.

### 3.4 Follicle development and reproductive organs

The effects of HRP powder supplementation on the reproductive organ characteristics of laying hens were presented in Table 6. Supplementation with 0.25%, 0.5%, and 0.75% HRP powder did not affect the relative weights of F1 follicles, non-F1 follicles, oviduct, infundibulum, magnum, isthmus, uterus, or vagina (P > 0.05). However, HRP supplementation linearly increased ovary weight (Linear, P = 0.03).

**Table 6:** Effects of dietary HRP powder supplementation on the morphology of reproductive organs and follicular development of laying hens (n = 5).

Variable	Group treatment				SEM	*p*-value (combined)	Contrasts	
	C0 (Control)	C1 (0.25%)	C2 (0.50%)	C3 (0.75%)			Linear	Quadratic
Ovary (%)	2.4	2.7	2.7	2.7	0.05	0.06	0.03	0.11
F1 folicle (%)	0.78	0,87	0.90	0.91	0.02	0.24	0.07	0.35
Non F1 folicles (%)	1.6	1.8	1.8	1.8	0.03	0.16	0.1	0.15
Oviduct (%)	4.1	4.0	4.1	3.8	0.13	0.90	0.65	0.75
Infundibulum (%)	0.11	0.13	0.10	0.11	0.01	0.63	0.69	0.76
Magnum (%)	2.0	2.0	2.2	1.8	0.07	0.26	0.64	0.20
Istmus (%)	0.37	0.35	0.34	0.39	0.01	0.61	0.58	0.25
Uterus (%)	1.1	1.2	1.1	1.1	0.03	0.72	0.56	0.36
Vagina (%)	0.26	0.41	0.38	0.39	0.03	0.37	0.22	0.30

Note: C0, basal diet; C1, basal diet + 0.25% hot red pepper powder; C2, basal diet + 0.50% hot red pepper powder; C3, basal diet + 0.75% hot red pepper powder. SEM, standard error of the mean.

### 3.5 Relative weight of digestive organ and digesta pH

Supplementation with 0.25%, 0.5%, and 0.75% HRP powder did not affect the relative weights of the liver, crop, proventriculus, gizzard, pancreas, duodenum, jejunum, ileum, colon, or caecum (P > 0.05; Table 7). Regarding digesta pH, HRP supplementation linearly decreased colon (Combined, P = 0.03; Linear, P = 0.02), and caecum (Combined, P < 0.01; Linear, P = 0.01; Quadratic, P = 0.03). However, no significant overall treatment effects were observed on the pH of the crop, duodenum, jejunum, and ileum (P > 0.05).

**Table 7:** Effects of dietary HRP powder supplementation on the digestive organs in laying hens (n = 5).

Variable	Group treatment				SEM	*p*-value (combined)	Contrasts	
	C0 (Control)	C1 (0.25%)	C2 (0.50%)	C3 (0.75%)			Linear	Quadratic
**Relative weight**								
Liver (%)	2.4	2.2	2.2	2.3	0.07	0.90	0.97	0.47
Crop (%)	0.60	0.67	0.70	0.70	0.03	0.68	0.26	0.64
Proventrikulus (%)	0.49	0.48	0.44	0.47	0.02	0.76	0.53	0.67
Gizzard (%)	1.4	1.7	1.6	1.5	0,47	0.22	0.76	0.09
Pancreas (%)	0.22	0.22	0.21	0.20	0.01	0.65	0.23	0.84
Duodenum (%)	0.47	0.49	0.46	0.47	0.01	0.88	0.85	0.73
Jejunum (%)	1.1	1.1	1.0	1.1	0.03	0.99	0.99	0.87
Ileum (%)	0.87	0.94	0.84	0.99	0.05	0.71	0.56	0.71
Colon (%)	0.29	0.27	0.29	0.32	0.01	0.43	0.25	0.23
Caecum (%)	0.51	0.52	0.52	0.52	0.03	1.0	0.99	0.86
**Digesta pH**								
Crop	5.0	4.9	4.8	4.8	0.29	0.64	0.30	0.54
Duodenum	6.2	6.2	6.0	5.9	0.33	0.48	0.13	0.89
Jejunum	6.2	6.3	5.9	5.9	0.32	0.16	0.09	0.98
Ileum	6.5	6.3	6.1	6.0	0.11	0.39	0.10	0.77
Colon	6.8^a^	6.5^a^	6.2^ab^	5.8^b^	0.12	0.03	0.02	0.10
Caecum	6.9^a^	6.5^ab^	5.9^c^	6.3^bc^	0.11	<0.01	<0.01	0.03

Note: a–c Different superscripts within the same row indicate significant differences (P < 0.05). C0, basal diet; C1, basal diet + 0.25% hot red pepper powder; C2, basal diet + 0.50% hot red pepper powder; C3, basal diet + 0.75% hot red pepper powder. SEM, standard error of the mean.

### 3.6 Jejunal histomorphology

The effects of dietary HRP powder on intestinal morphology of laying hens were presented in Table 8 and Fig. 4. Supplementation with 0.25%, 0.5%, and 0.75% HRP powder did not affect villus height and villus-to-crypt ratio (P > 0.05). However, dietary HRP powder linearly increased villus width (Combined, P = 0.05; Linear, P = 0.02; Quadratic, P = 0.41), villus surface area (Combined, P = 0.05; Linear, P = 0.01; Quadratic, P = 0.97), and crypt depth (Combined, P = 0.18; Linear, P = 0.04; Quadratic, P = 0.81).

**Table 8:** Effects of dietary HRP powder supplementation on the histomorphology of the jejunum in laying hens (n = 5).

**Variable**	**Group Treatment**				**SEM**	**p-value (combined)**	**Regression**	
	**C0 (control)**	**C1 (0.25%)**	**C2 (0.50%)**	**C3 (0.75%)**			**Linear**	**Quadratic**
Villus height (μm)	910	880	921	976	6.2	0.85	0.51	0.59
Villus width (μm)	75.4	77.8	92.4	87.1	24.4	0.05	0.02	0.41
Villus surface area (× 10^3^ μm^2^)	106	105	133	133	3.5	0.05	0.01	0.97
Crypt depth (μm)	219	214	218	235	4.3	0.18	0.04	0.81
Villus: crypt	4.18	4.30	4.34	4.17	0.14	0.75	0.39	0.84

Note: a–c Different superscripts within the same row indicate significant differences (P < 0.05). C0, basal diet; C1, basal diet + 0.25% hot red pepper powder; C2, basal diet + 0.50% hot red pepper powder; C3, basal diet + 0.75% hot red pepper powder. SEM, standard error of the mean.

**Fig. 4:**
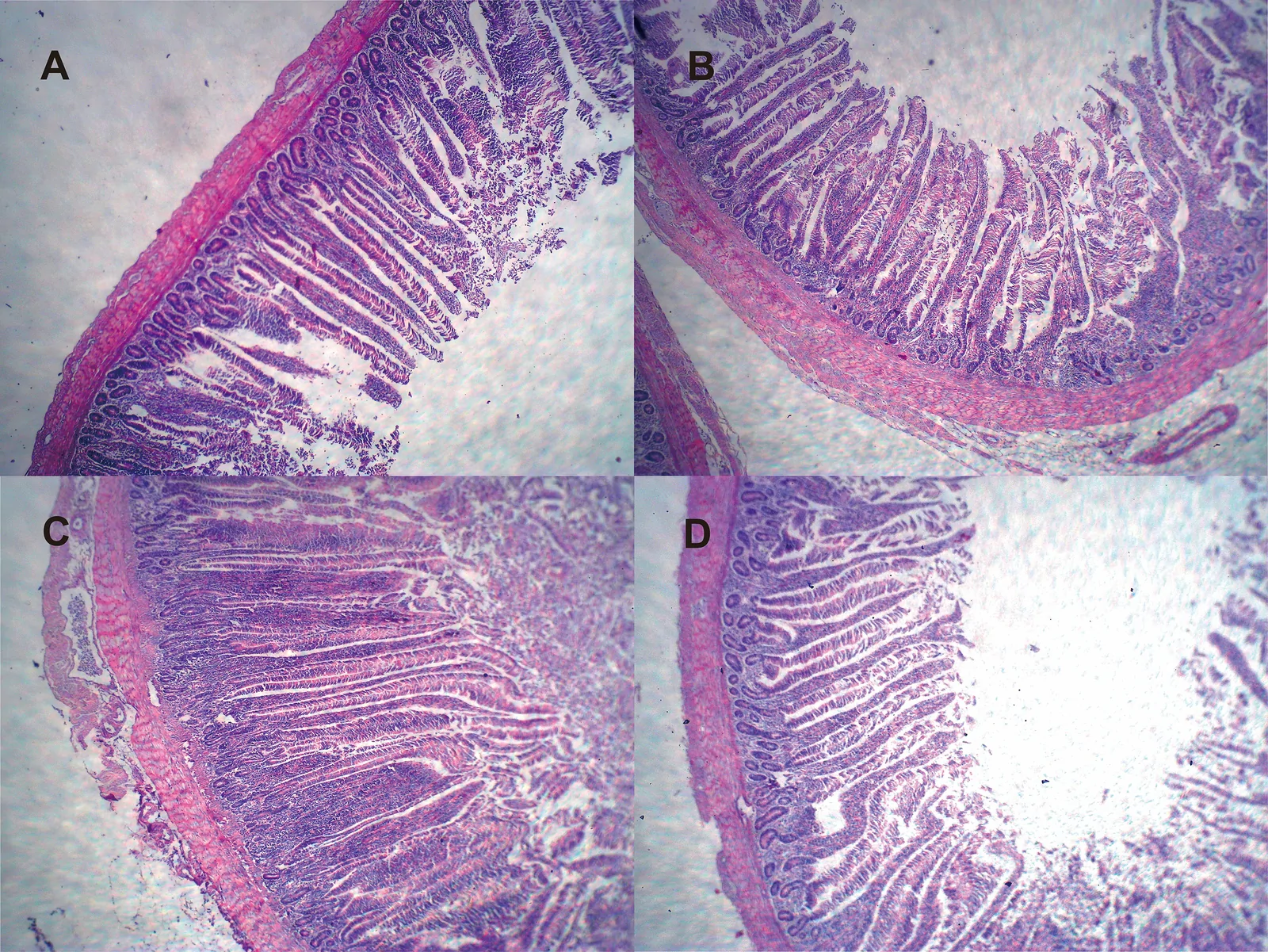
Representative jejunal histomorphology of laying hens following dietary supplementation with HRP powder (10 × magnification; hematoxylin and eosin staining): (A) control group (basal diet); (B) 0.25%; (C) 0.50%; and (D) 0.75% HRP supplementation.

### 3.7 Liver histopathology and antioxidant capacity

The effects of HRP powder supplementation on oxidative stress biomarkers in laying hens were presented in Fig. 5**.** Supplementation with 0.25%, and 0.5% HRP powder increased superoxide dismutase (SOD) activity (Combined, P = 0.04; Linear, P = 0.34; Quadratic, P = 0.008). In addition, dietary HRP powder 0.5–0.75% decreased malondialdehyde (MDA) concentration (Combined, P = 0.03; Linear, P < 0.01; Quadratic, P = 0.43).

**Fig. 5:**
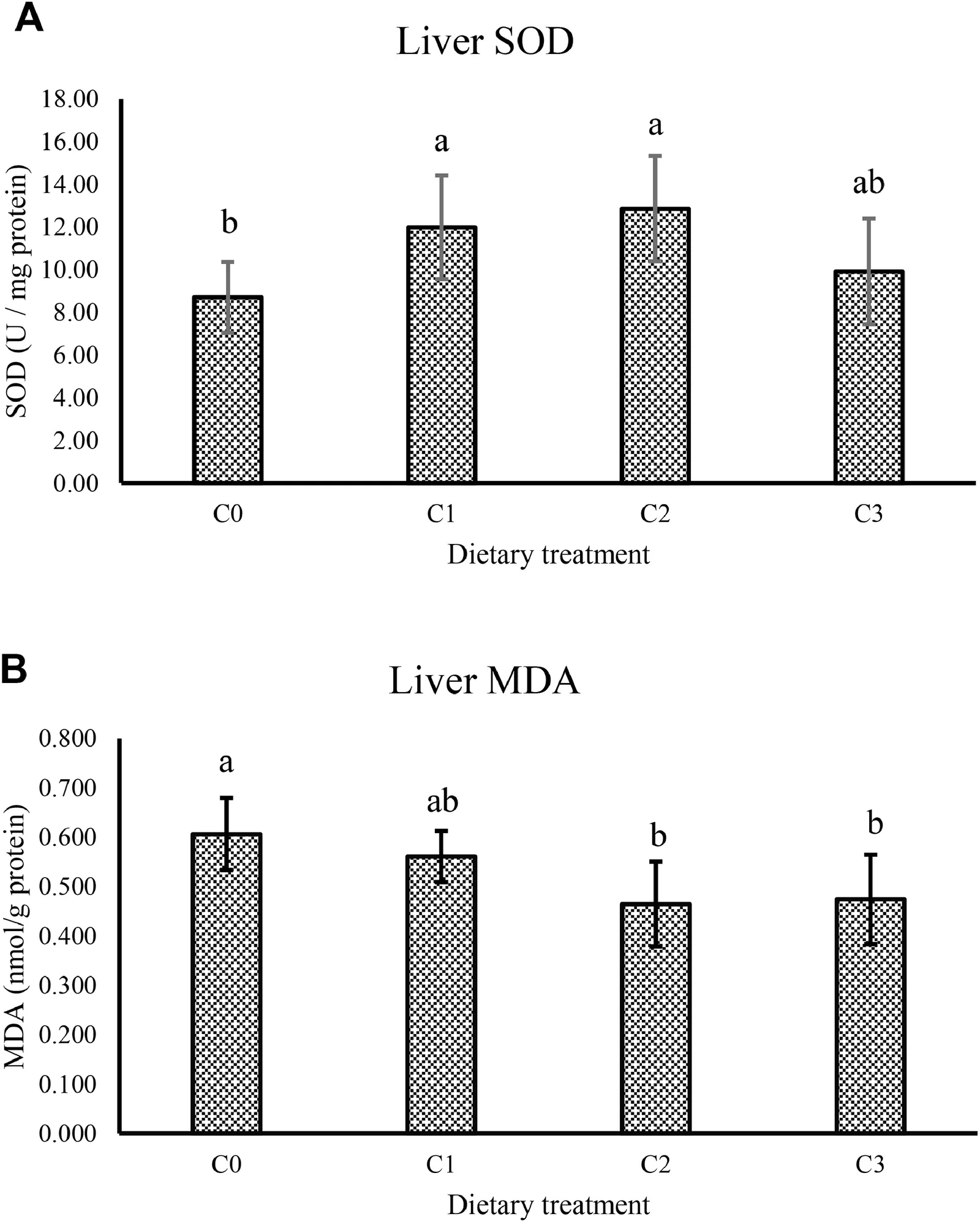
Effects of dietary hot red pepper powder supplementation on the liver antioxidant activity in laying hens: (A) superoxide dismutase activity; (B) malondialdehyde concentration.

**Note:** C0, basal diet; C1, basal diet + 0.25% HRP powder; C2, basal diet + 0.5% HRP powder; C3, basal diet + 0.75% HRP powder; a–b Different superscripts within the same row indicate significant differences (P < 0.05).

Histopathological observation of the liver at 10 × magnification showed pronounced inflammatory infiltration in the portal area of the control group (Fig. 6**A**), whereas the C1, C2, and C3 groups showed only mild inflammatory features (Fig. 6**B–D**). At 40 × magnification, hepatocellular vacuolar degeneration was observed in the control group (Fig. 7**A**). The C1 and C3 groups showed less prominent but still observable vacuolar degeneration (Fig. 7**B and D**), while the C2 group showed relatively fewer observable vacuolated hepatocytes (Fig. 7**C**).

**Fig. 6:**
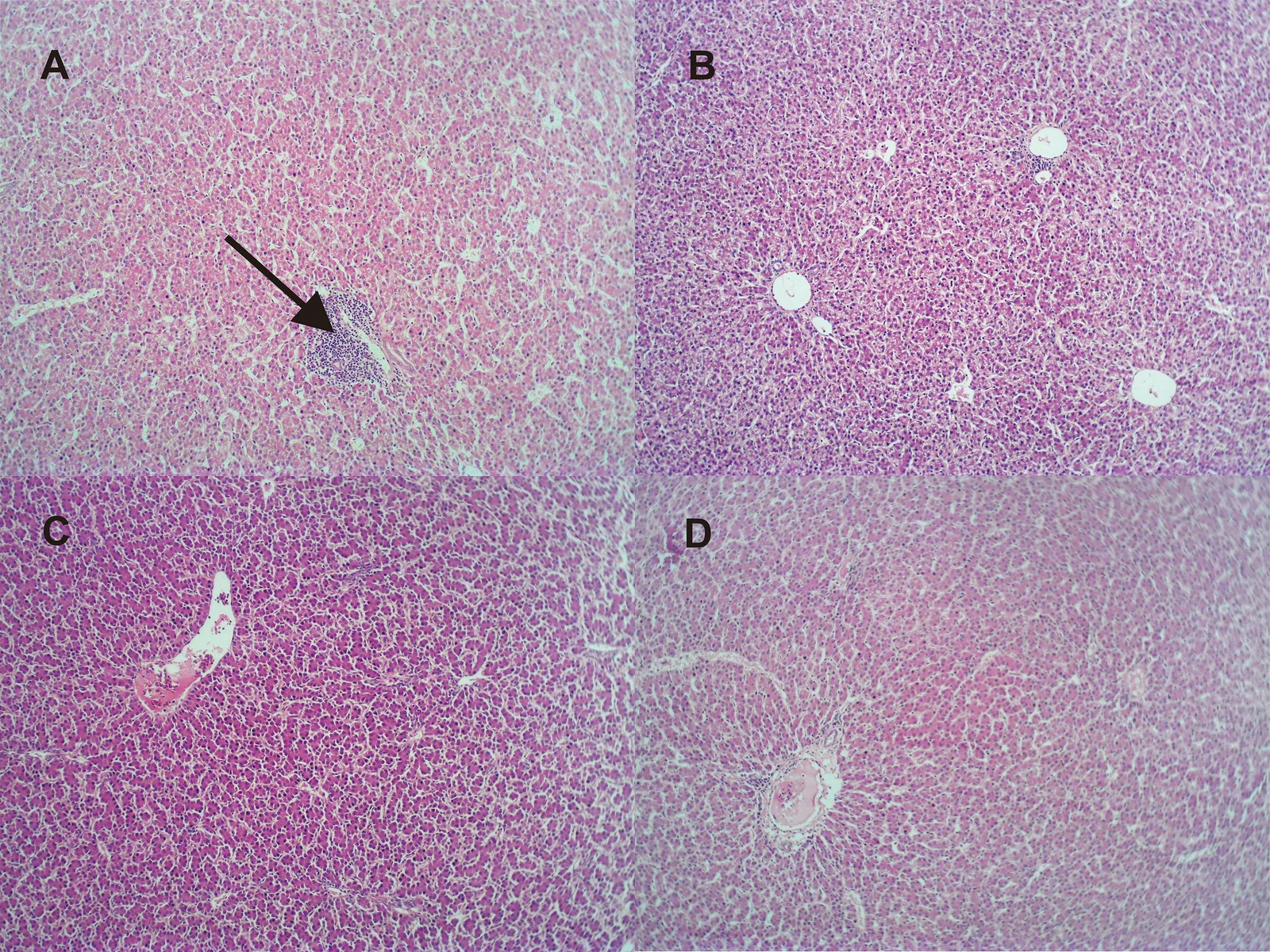
Liver histopathological changes in laying hens following dietary supplementation with hot red pepper powder (10 × magnification; hematoxylin and eosin staining): (A) basal diet (control); (B) 0.25%; (C) 0.50%; and (D) 0.75% HRP powder.

**Fig. 7:**
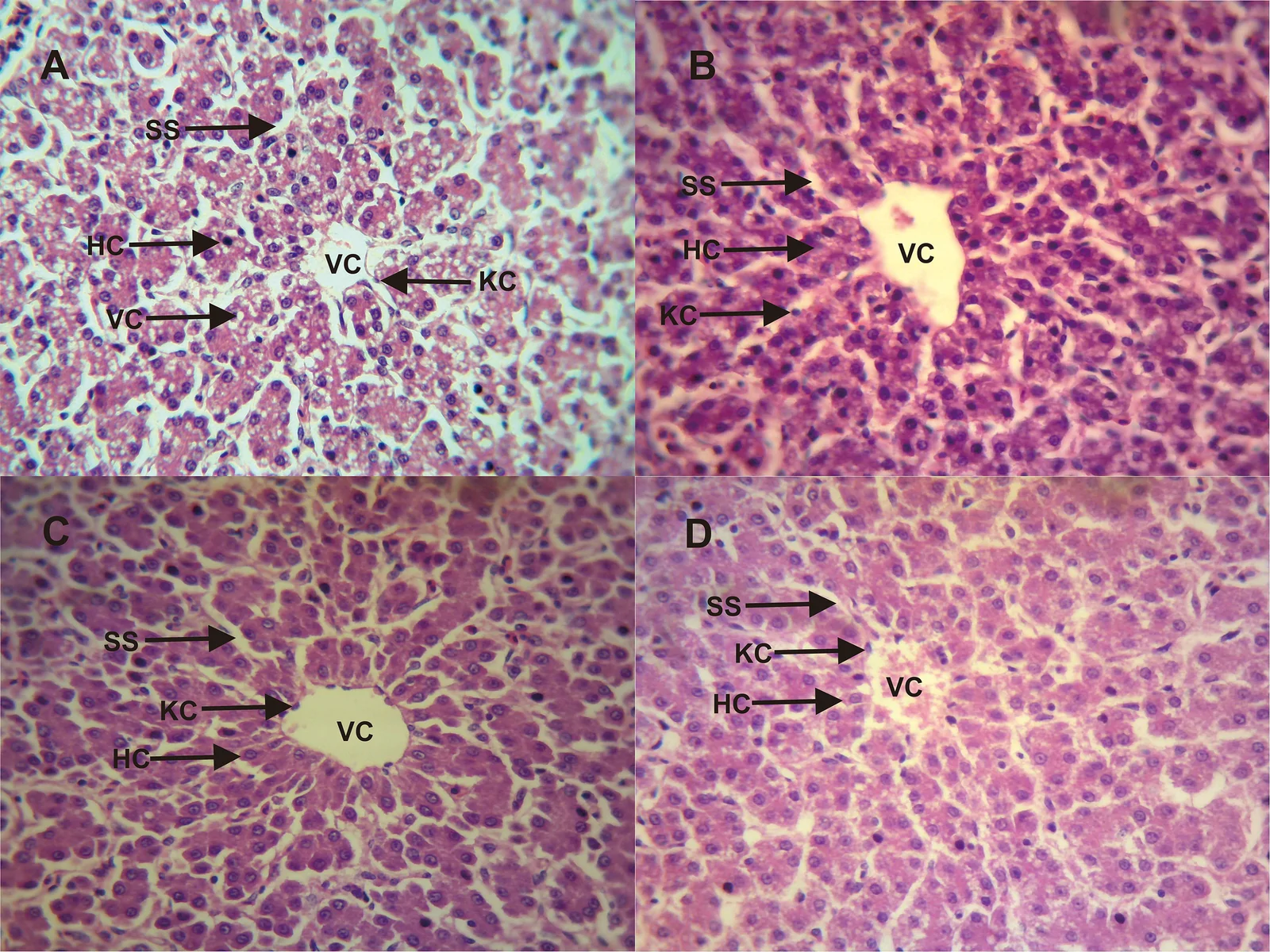
Liver histopathology of laying hens following dietary supplementation with hot red pepper powder (40 × magnification; hematoxylin and eosin staining): (A) basal diet (control); (B) 0.25%; (C) 0.50%; and (D) 0.75% HRP supplementation.

Note: VC=vena centralist, HC=hepatocyte, KC=Kupffer cells, SS=sinusoid, VD: Vacuolar Degeneration.

## 4 Discussion

HRP powder supplementation did not affect feed intake in laying hens, indicating that the inclusion levels used in the present study did not alter feed consumption. This finding is consistent with previous studies reporting no effect of red pepper or HRP powder supplementation at levels of 0.4–0.8% on feed intake (Abou-Elkhair et al., 2018; Aderemi et al., 2013; Lokaewmanee et al., 2013; Li et al., 2012). In contrast, reduced feed intake has been reported at higher supplementation levels of 1–1.5% (Aikpitanyi & Imasuen, 2023), while a meta-analysis by Hanif et al. (2024) suggested that pepper powder supplementation above 0.9% may reduce feed intake. Therefore, the absence of an effect in the present study may indicate that the HRP inclusion levels were within a range that did not adversely affect feed intake.

Despite the unchanged feed intake, supplementation with 0.25 and 0.5% HRP increased daily egg production. This finding suggests that the increase in egg production occurred without a corresponding increase in feed consumption. Similar responses have been reported with moderate levels of red pepper supplementation, although the response has not been consistent across studies (Abou-Elkhair et al., 2018; Aderemi et al., 2013; Lokaewmanee et al., 2013). The inconsistent findings among studies may be related to differences in supplementation level, duration, bird age, and experimental conditions.

HRP powder supplementation did not affect egg weight, indicating that HRP supplementation did not alter individual egg size under the conditions of the present study. This finding is consistent with previous studies reporting no effect of red pepper or HRP supplementation on egg weight at different inclusion levels (Abou-Elkhair et al., 2018; Aderemi et al., 2013; Moeini et al., 2013). Abou-Elkhair et al. (2018), however, reported an increase in egg weight during the second experimental period, suggesting that the response may differ among experimental conditions and treatment periods. In contrast, HRP supplementation increased egg mass linearly. Because egg mass is determined by egg production and egg weight, the increase in egg mass in the present study may be mainly related to the increase in egg production, given that egg weight was not affected by HRP supplementation.

Previous studies have reported inconsistent effects of red pepper supplementation on egg mass, with increases observed by Abou-Elkhair et al. (2018), but no significant effects reported by Moeini et al. (2013) and Bala et al. (2020). Furthermore, dietary HRP powder supplementation did not significantly alter the income over feed cost (IOFC), indicating that the observed biological improvements did not translate into a clear economic advantage under the present experimental conditions. Although mean IOFC values were numerically lower in the HRP-supplemented groups, these differences were not statistically significant. IOFC is commonly used to assess the economic outcome of poultry production because it integrates both income from egg production and feed expenditure (Pradana et al., 2025). Variations in feed ingredient composition, feed cost, and daily feed intake typically drive differences in IOFC outcomes (Katu et al., 2025). Therefore, practical recommendations for HRP supplementation must acknowledge that economic benefits were not demonstrated in this trial, and future studies should evaluate diet optimization to align biological efficacy with financial viability

HRP powder supplementation did not significantly alter serum total protein, albumin, urea, blood urea nitrogen (BUN), or uric acid concentrations in laying hens, indicating that nitrogen metabolism and key serum protein parameters remained unaffected under the current experimental conditions. These findings align with Moeini et al. (2013), who observed no changes in urea or uric acid at red pepper levels up to 3%. In contrast, Aikpitanyi and Imasuen (2024) reported altered protein parameters at higher inclusion levels (1–1.5%), suggesting that variations across studies stem primarily from differences in supplementation levels and experimental designs. overall, HRP supplementation at the tested levels exerted no adverse or significant influence on the evaluated serum protein and nitrogen markers.

Dietary HRP supplementation at 0.25%, 0.5%, and 0.75% significantly reduced serum cholesterol concentrations without affecting triglyceride levels. This finding is consistent with previous studies reporting cholesterol-lowering effects of red pepper or HRP supplementation in poultry (Abou-Elkhair et al., 2018; Aikpitanyi & Imasuen, 2024; Puvača et al., 2015), although Moeini et al. (2013) reported reduced triglyceride levels without changes in cholesterol. Such variation may reflect differences in inclusion levels, supplementation duration, and experimental conditions. Although the precise mechanism cannot be determined from the present data, the cholesterol-lowering effect may be associated with the bioactive compounds in HRP. Capsaicin may modulate lipid metabolism through transient receptor potential vanilloid 1 (TRPV1) activation, which has been linked to enhanced cholesterol efflux and reduced lipid accumulation (Ma et al., 2011; Zhang et al., 2025a, 2025b). In addition, phenolic compounds may inhibit cholesterol synthesis by modulating 3‑hydroxy-3-methylglutaryl-coenzyme A activity and lipid utilization (Akbarian et al., 2011; Sato, 2020; Rodríguez-Pérez et al., 2019; Zeng et al., 2025), while saponins may reduce intestinal cholesterol absorption through binding mechanisms (Chávez-Santoscoy et al., 2013; Chen et al., 2017).

In this study, HRP supplementation significantly altered the leukocyte profile, characterized by decreased total leukocyte (LEU) and heterophil (HET) counts and increased lymphocyte (LYM) counts. These changes are consistent with previous reports of reduced HET and increased LYM following red pepper supplementation in poultry (Aikpitanyi & Imasuen, 2024; Prieto & Campo, 2010). Although bioactive compounds such as capsaicin may modulate immune and inflammatory pathways (Kim et al., 2014; Li et al., 2023; Zhang et al., 2023). The reduction in HET and increase in LYM were accompanied by a significantly lower heterophil-to-lymphocyte (H/L) ratio, a commonly used indicator of physiological stress in poultry (Minias, 2019; Thiam et al., 2022; Taghizadeh et al., 2024). Because heterophils contribute primarily to innate immunity, whereas lymphocytes are central to adaptive immunity, this shift may indicate a less acute immune response or reduced physiological stress in the laying hens (Chen et al., 2026; Kogut, 2022).

Dietary HRP supplementation resulted in a linear increase in ovarian weight. This finding is consistent with previous studies reporting increased ovarian or follicular weight following red pepper or capsaicin supplementation in laying birds (Korkmaz et al., 2023; Özer et al., 2005; Liu et al., 2021a). The increase in ovarian weight may indicate a response of ovarian tissue to HRP supplementation; however, the underlying mechanisms cannot be determined from the present data. Previous studies have suggested that capsaicin, vitamins, and other bioactive compounds may influence ovarian function by modulating follicular development, antioxidant status, and reproductive signaling (Attia et al., 2016; Güler & Zik, 2018; Sahin et al., 2017; Wang et al., 2026; Zik et al., 2010).

HRP powder supplementation did not affect the relative weights of the liver, gizzard, or pancreas in laying hens. The absence of an effect on liver weight is consistent with previous findings in broilers receiving 0.2–0.4% red pepper powder (Al-Doori et al., 2024), although a reduction in liver weight has been reported at a higher inclusion level of 1% (Mudalal & Badran, 2025). Similarly, the present findings for gizzard and pancreas weights are generally consistent with previous studies reporting no significant effects of red pepper supplementation on internal organ weights (Al-Doori et al., 2024; Mudalal & Badran, 2025). HRP supplementation also did not affect the relative weights or morphology of the measured digestive tract segments, including the crop, proventriculus, duodenum, jejunum, ileum, and cecum. This indicates that the tested HRP levels did not significantly alter the measured digestive tract characteristics under the conditions of the present study. In contrast, changes in intestinal weight have been reported following supplementation with Brazilian red pepper oil (Silva et al., 2011; Xiang et al., 2021, 2022).

HRP supplementation also reduced large-intestinal and cecal digesta pH, indicating improved modulation of the hindgut environment. Similar findings have been reported following supplementation with red pepper oil or Capsicum extract, which reduced cecal pH and improved microbial health in chickens (Angkanaporn et al., 2024; Arisha et al., 2024; Hassan et al., 2023; Reda et al., 2020). These effects are likely mediated by capsaicin, which can enhance beneficial microbial populations, improve antioxidant and anti-inflammatory status, and promote fermentation processes in laying hens (Zhang et al., 2025a, 2025b). The reduced hindgut pH observed in this study may reflect increased production of short-chain fatty acids (SCFAs), microbial metabolites associated with improved gut health, nutrient utilization, and intestinal barrier function (Liu et al., 2021b; Madani et al., 2024; Onrust et al., 2020). In addition, carotenoids and other phytochemicals present in HRP may further support gut microbial balance and SCFA production, contributing to enhanced digestive and immune function in laying hens (Guo et al., 2019; Hui et al., 2020), although individual SCFA profiles and microbial populations were not directly measured in the present work.

HRP powder supplementation did not produce significant overall treatment effects on jejunal histomorphology. These results align with previous observations of unchanged villus height and crypt morphology following red pepper or capsaicin supplementation in laying hens (Lokaewmanee et al., 2013; Xiao et al., 2025), contrasting with the reduced crypt depth reported only at higher capsaicin doses (Zhang et al., 2025b). However, increasing HRP supplementation levels resulted in significant linear responses in jejunal villus width and total villus surface area, indicating progressive changes in these morphological parameters that may contribute to the functional absorptive capacity of the jejunum and nutrient absorption (El Sabry & Abd El-Ghany, 2021; Simon et al., 2019). This structural enhancement supports findings by Lokaewmanee et al. (2013), who noted increased jejunal cell area with 0.5% pepper supplementation, and Li et al. (2022), who observed increased villus width in broilers fed capsaicin at 2 mg/kg.

In this study, marked portal inflammatory infiltration was observed in the control group, whereas the groups supplemented with HRP powder (0.25%, 0.50%, and 0.75%) showed mild inflammatory infiltration. The temperature–humidity index (THI) indicated moderate thermal stress during rearing, which likely triggered these hepatic alterations; heat stress is well-documented to induce portal inflammatory cell infiltration, promote oxidative stress, and impair immune function (Moraes et al., 2008; Nawab et al., 2019; Robinson et al., 2016). Because approximately 70–80% of hepatic blood supply originates from the gastrointestinal tract via the portal vein, the liver experiences a heavy immunological burden from gut-derived antigens, toxins, and microbial metabolites (Kubes & Jenne, 2018; Robinson et al., 2016). Within this immune surveillance axis, heterophils act as frontline responders that execute phagocytosis, cytokine production, and tissue repair (Kogut, 2022). Histological examination (40 ×) further revealed hepatocellular vacuolar degeneration in control livers (Wilk et al., 2023). HRP supplementation attenuated the severity of these vacuolar lesions at 0.25% and 0.75%, with the 0.50% inclusion level yielding the most pronounced histological protection.

The hepatoprotective effects of capsaicin are primarily driven by its ability to attenuate oxidative stress, inflammation, and apoptosis. Capsaicin enhances antioxidant enzyme activities—such as superoxide dismutase (SOD), catalase (CAT), and glutathione S-transferase (GST)—while decreasing malondialdehyde (MDA) levels (Doğan et al., 2022; Hassan et al., 2012; Karimi-Sales et al., 2024; Li et al., 2024). Concurrently, it suppresses hepatic inflammatory mediators, while diminishing macrophage infiltration (Li et al., 2023; Nunes et al., 2025). Mechanistically, capsaicin acts through TRPV1 activation to modulate thermoregulation, lipid metabolism, mitochondrial function, and circadian rhythm (Bujak et al., 2019; Sukmanadi et al., 2025). Additionally, capsaicin-mediated activation and peroxisome proliferator-activated receptor pathways reduces hepatic triglyceride accumulation, further alleviating liver inflammation (Li et al., 2013).

In the present study, dietary HRP powder supplementation significantly enhanced hepatic SOD activity (at 0.25% and 0.50% inclusion levels) and reduced hepatic MDA concentrations (at 0.50% and 0.75% levels) in laying hens. This combined response demonstrates a clear mitigation of hepatic lipid peroxidation alongside a reinforced enzymatic antioxidant defense. The observed biological effects are primarily attributable to capsaicin, the principal bioactive alkaloid in HRP, which is known to enhance systemic and tissue antioxidant capacity (Liu et al., 2021a). Mechanistically, capsaicin can scavenge reactive oxygen species (ROS) directly and is widely reported to upregulate SOD enzyme activity and hepatic SOD1 gene expression (Kreuz et al., 2022). Furthermore, capsaicin-mediated reductions in MDA levels are potentially linked to the activation of the Nrf2 signaling pathway—which drives antioxidant enzyme transcription—and the downregulation of pro-inflammatory cascades such as NF-κB, thereby protecting hepatic membranes from oxidative degradation (Elsherif et al., 2019; Hu et al., 2025). In addition to capsaicin, the phenolic and flavonoid constituents of HRP likely act synergistically by providing hydrogen-donating capacity to terminate lipid peroxidation chains (Srinivasan, 2016; Surai et al., 2019)

The MDA reduction aligns with previous poultry studies reporting lowered systemic, ovarian, and intestinal MDA levels following red pepper or capsaicin intake (Abed & Al-Shukri, 2024; Abou-Elkhair et al., 2018; Fan et al., 2023; Liu et al., 2021a; Xiao et al., 2025), though null effects have occurred at excessive inclusion levels (Bala et al., 2020). Mechanistically, capsaicin mitigates lipid peroxidation by downregulating inflammatory cascades that aggravate hepatic oxidative damage (Hu et al., 2025; Liu et al., 2021a). Furthermore, red pepper polyphenols likely contribute synergistically to this response, as plant polyphenols consistently depress MDA concentrations while elevating antioxidant defenses in laying hens (Chen et al., 2018; Ding et al., 2023; Yuan et al., 2025). Overall, this MDA decline reflects enhanced antioxidant capacity and improved physiological resilience (Surai et al., 2019).

## 5 Conclusion

In conclusion, HRP supplementation at 0.25–0.5% improved early Hen-Day Production, while inclusion at 0.25–0.75% consistently reduced serum cholesterol and the H/L ratio. A 0.5% HRP inclusion level was associated with higher hepatic SOD activity and lower MDA concentration, together with relatively favorable descriptive histopathological observations, indicating potential antioxidant and hepatoprotective effects. Additionally, HRP enhanced gut morphology and microenvironment by increasing jejunal villus width and lowering cecal and colonic pH. Consequently, HRP powder, particularly at 0.5%, holds strong potential as a natural dietary strategy to improve production performance, systemic health, and oxidative status in laying hens. However, this study did not evaluate key egg quality traits nor did it characterize gut bacterial diversity or short-chain fatty acid (SCFA) profiles. Future research incorporating comprehensive egg quality analyses alongside microbiome sequencing and metabolite profiling is recommended to fully elucidate the nutritional and physiological impacts of HRP supplementation.

## Declaration of generative AI and AI-assisted technologies in the writing process

During the preparation of this manuscript, the authors used ChatGPT (OpenAI) solely for language translation and linguistic assistance. The tool was not used to generate scientific content, formulate research hypotheses, analyze or interpret data, or draw scientific conclusions. All translated and AI-assisted text was carefully reviewed, edited, and validated by the authors. The authors take full responsibility for the content, accuracy, integrity, and originality of the manuscript.

## Statement for animal studies

All animal procedures were conducted in accordance with relevant institutional guidelines and regulations for the care and use of animals in research. The study protocol was reviewed and approved by the Research Ethics Committee of the Faculty of Veterinary Medicine, Universitas Gadjah Mada, Yogyakarta, Indonesia (Ref. No. 98/EC-FKH/Eks/2023).

## CRediT authorship contribution statement

**Muhammad Fathin Hanif:** Writing – review & editing, Writing – original draft, Visualization, Resources, Methodology, Investigation, Formal analysis, Data curation. **Ali Agus:** Writing – review & editing, Supervision, Resources, Funding acquisition, Conceptualization. **Bambang Ariyadi:** Writing – review & editing, Supervision, Conceptualization. **Muhlisin:** Writing – review & editing, Supervision, Conceptualization. **Nanung Danar Dono:** Writing – review & editing, Conceptualization. **Heru Sasongko:** Writing – review & editing, Conceptualization. **Chusnul Hanim:** Writing – review & editing, Conceptualization.

## Declaration of competing interest

All authors declare that there are no conflicts of interest regarding the publication of this article.
